# Simultaneous Down-Regulation of Intracellular hTERT and GPX4 mRNA Using MnO_2_-Nanosheet Probes to Induce Cancer Cell Death

**DOI:** 10.3390/s26030836

**Published:** 2026-01-27

**Authors:** Yixin Miao, Tao Zhou, Qinghong Ji, Min Hong

**Affiliations:** School of Chemistry and Chemical Engineering, Liaocheng University, Liaocheng 252059, China; 18866211076@163.com (Y.M.); 19560850686@163.com (T.Z.); 17861826032@163.com (Q.J.)

**Keywords:** manganese dioxide nanosheet probe, hTERT, GPX4, gene silencing, inhibiting cancer cell proliferation

## Abstract

Cancer remains a leading global cause of death, with conventional treatments often limited by toxicity and recurrence. Recent advances in gene therapy and nanodrug delivery offer new avenues for precision oncology. Human telomerase reverse transcriptase (hTERT) and glutathione peroxidase 4 (GPX4) are overexpressed in many cancers and linked to apoptosis and ferroptosis, respectively. Here, we developed a manganese dioxide nanosheet (MnO_2_-NS) probe co-loaded with antisense oligonucleotides targeting hTERT and GPX4 mRNA to synergistically down-regulate both genes and induce dual cell death pathways. The probe, assembled via adsorption of fluorescently labeled antisense strands, showed controllable release in the presence of glutathione (GSH). Cellular uptake and antisense release were confirmed in multiple cancer cell lines. The MnO_2_-NS probe significantly suppressed cell proliferation, outperforming single-target or carrier-only controls. Molecular analyses confirmed reduced hTERT and GPX4 expression, along with GSH depletion, ROS accumulation, and elevated lipid peroxidation—collectively promoting enhanced cancer cell death. In summary, this MnO_2_-NS-based co-delivery system enables synergistic gene silencing and GSH depletion, enhancing antitumor efficacy and providing a promising strategy for multifunctional nanotherapy.

## 1. Introduction

Cancer remains one of the leading causes of human mortality worldwide. Conventional treatment strategies—such as surgery, radiotherapy, and chemotherapy—often suffer from limitations including systemic toxicity, incomplete efficacy, and inability to prevent recurrence. In recent years, early diagnosis and personalized precision medicine have emerged as pivotal approaches to overcoming cancer. With the successive identification of numerous “oncogenes” and “tumor suppressor genes,” gene therapy has shown considerable promise in oncology. Molecular profiling—based on molecular biological differences during disease onset and progression—enables the identification of key biomolecules (e.g., proteins and nucleic acids) as therapeutic targets. This facilitates targeted drug delivery and personalized treatment, improving therapeutic accuracy. By acting specifically on cancer cells, molecularly targeted gene therapies can effectively kill malignant cells while minimizing damage to normal tissues, thereby reducing patient suffering and offering renewed hope for survival. Since its introduction, tumor gene therapy has achieved remarkable clinical outcomes, gaining broad recognition and establishing itself as a highly promising modality for individualized cancer treatment [1].

Human telomerase reverse transcriptase (hTERT), the catalytic subunit of telomerase, plays a critical role in maintaining telomere length and enabling cellular immortalization. Down-regulation of hTERT induces telomere shortening and dysfunction, activating p53-dependent or independent DNA damage response pathways. This leads to loss of mitochondrial membrane potential, cytochrome c release, caspase cascade activation, and ultimately, apoptosis [2,3]. In parallel, glutathione peroxidase 4 (GPX4) serves as a key membrane lipid repair enzyme. Knockout of GPX4 results in rapid accumulation of lipid peroxide (LPO), particularly in cell membranes, compromising membrane integrity and causes cell death in a pathologically relevant form of ferroptosis [4,5]. Recent studies have shown that simultaneously inducing both apoptosis and ferroptosis in cancer cells through different means can produce a more potent anti-tumor effect [6,7]. Specifically, interfering with the levels of hTERT and GPX4 within cancer cells is expected to kill cancer cells more effectively by leveraging the synergy between apoptosis and ferroptosis, thereby achieving a highly efficient anti-tumor outcome.

Antisense oligonucleotide (ASO) technology has been shown to effectively down-regulate the expression of both hTERT [8] and GPX4 mRNAs [9] thereby reducing intracellular levels of these proteins. Studies indicate that individually targeting hTERT or GPX4 promotes modest levels of apoptosis or ferroptosis, respectively; however, the overall suppression of cancer cell proliferation remains limited and insufficient to eradicate tumors completely. We hypothesize that simultaneous down-regulation of both genes may induce synergistic tumoricidal effects: hTERT inhibition activates intrinsic apoptotic pathways, while GPX4 suppression disrupts lipid peroxide repair, unlocking the ferroptosis pathway. This “dual-hit” strategy is expected to accelerate cancer cell death more effectively than single-target approaches.

Manganese dioxide (MnO_2_) nanomaterials (e.g., hollow MnO_2_ nanoparticles (MnO_2_-NPs) and MnO_2_ nanosheets (MnO_2_-NSs)) have been extensively investigated for antitumor applications [10]. In the tumor microenvironment, these materials participate in redox reactions with glutathione (GSH, a major antioxidant), leading to its depletion and consequently compromising the cellular antioxidant defense system. For instance, asymmetrical Au@MnO_2_ nanomotors generate oxygen via asymmetric catalytic reactions, which facilitates Fenton-like reactions that release Mn^2+^ ions while simultaneously consuming GSH [11]. The depletion of GSH weakens the antioxidant capacity of tumor cells, thereby enhancing the cytotoxic effects of reactive oxygen species (ROS) and ultimately inducing apoptosis or necrosis. Furthermore, the released Mn^2+^ ions activate the cGAS-STING signaling pathway, promoting dendritic cell maturation and T-cell infiltration, which collectively contribute to reversing the immunosuppressive tumor microenvironment [12,13].

**Figure 1:**
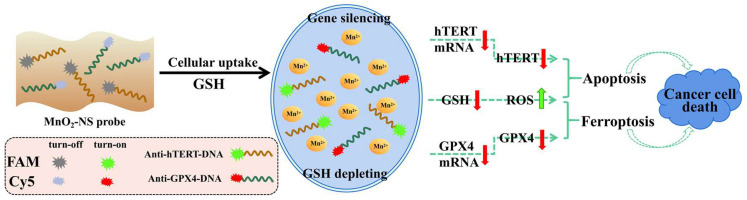
Schematic illustration of designation of MnO_2_-NS probes, mechanism of inducing cancer cell death by the synergism of GSH depletion and down-regulation of hTERT and GPX4 simultaneously through gene silencing.

Based on the loading capacity of MnO_2_-NSs for single-stranded oligonucleotides [14,15], here we designed a MnO_2_-NS probe co-loaded with ASOs targeting hTERT and GPX4 mRNAs to verify the synergistic role of MnO_2_-NSs in enhancing anticancer efficacy by depleting intracellular GSH while concurrently down-regulating hTERT and GPX4 expression (Figure 1).

## 2. Materials and Methods

### 2.1. Reagents

Manganese(II) chloride tetrahydrate (MnCl_2_·4H_2_O), tetramethylammonium hydroxide (TMA·OH), GSH, dimethyl sulfoxide (DMSO), N-(3-dimethylaminopropyl)-N′-ethylcarbodiimide hydrochloride (EDC·HCl), N-hydroxysuc-cinimide (NHS), β-mercaptoethanol, and 2-morpholin-4-yl ethane sulfonic acid hydrate (MES), and 3-(4,5-dimethylthiazol-2-yl)-2,5-diphenyltetrazolium bromide (MTT) were obtained from Aladdin Reagent Co., Ltd. (Shanghai, China). Hydrogen peroxide (H_2_O_2_, 3 wt%) and phosphate-buffered saline (PBS, pH 7.4, 136.7 mM NaCl, 2.7 mM KCl, 8.72 mM Na_2_HPO_4_, and 1.41 mM KH_2_PO_4_) were used in the study. All aqueous solutions were prepared using ultrapure water. The DNA sequences used (Table 1) was synthesized and purchased from Sangon Biotechnology Co., Ltd. (Shanghai, China).

### 2.2. Synthesis and Characterization of MnO_2_-NSs

MnO_2_-NSs were synthesized according to the method reported previously [16]. Briefly, rapidly mix a 20 mL mixture of TMA·OH (0.6 M) and H_2_O_2_ (3 wt%) with 10 mL of MnCl_2_·4H_2_O (0.3 M) solution within 15 s to form a deep brown suspension. The resulting mixture was stirred at room temperature for approximately 24 h. The product was then collected by centrifugation at 2000 rpm for 10 min, washed with deionized water and methanol, and dried at −60 °C. Subsequently, 10 mg of the dried crude product was dispersed in 20 mL of PBS solution and subjected to ultrasonication for 10 h, and the final solution was kept in refrigerator (4 °C) for use in the following experiment. The experimental measurements were conducted using a JEM-2100 transmission electron microscope (JEOL, Tokyo, Japan), a MultiMode 8 atomic force microscope (Bruker, Santa Barbara, CA, USA), and a 90Plus/BI-MAS instrument (Brookhaven Instruments, Carlsbad, CA, USA) for particle size and morphology analysis. The absorption spectra of MnO_2_-NSs was measured using a UV-750 ultraviolet spectrophotometer (Perkin-Elmer, Waltham, MA, USA).

### 2.3. Preparation of MnO_2_-NS-Based Probes

Anti-hTERT-DNA (5 µM, 1 mL) and Anti-GPX4-DNA (5 µM, 1 mL) were sequentially added to the MnO_2_-NS (8 mL, 0.5 mg/mL) solution. The resulting solution after the mixture reacting for 24 h was centrifuged for 20 min (10,000 rpm) in a centrifuge, and then washed three times with deionized water. Then the final precipitation was solved in PBS (8 mL) solution and mixed for 2 h to obtain the Anti-hTERT/GPX4-MnO_2_-NS probes. The concentration of MnO_2_-NSs was evaluated using an Inductively Coupled Plasma Mass Spectrometer (ICP-MS, iCAP RQ, Thermo Scientific, Waltham, MA, USA).

Anti-hTERT-MnO_2_-NS probes, Anti-GPX4-MnO_2_-NS probes, and Control-MnO_2_-NS probes were prepared with the similar procedure mentioned above but incubated MnO_2_-NS (8 mL, 0.5 mg/mL) with Anti-hTERT-DNA (5 µM, 2 mL), Anti-GPX4-DNA (5 µM, 2 mL), or Control-DNA (5 µM, 2 mL), respectively.

### 2.4. Determination of Fluorescence Quenching and Recovery

Fluorescence quenching assay: Different amounts of MnO_2_-NSs solution (final concentrations: 0, 20, 40, 60, 80, and 100 μg/mL) were mixed with Anti-hTERT-DNA (final concentration: 100 nM) and Anti-GPX4-DNA (final concentration: 100 nM). The mixture was then diluted with PBS buffer to a final volume of 200 μL. After 30 min, the fluorescence intensity of each sample was recorded using a Hitachi F-7000 fluorospectrometer (Tokyo, Japan) (excitation wavelengths: 488 nm and 633 nm).

Fluorescence restoration assay: Different concentrations of GSH (0, 1, 2, 4, and 10 mM) were mixed with the Anti-hTERT/GPX4-MnO_2_-NS probe (40 μg/mL). The mixture was diluted with PBS buffer to a final volume of 200 μL. After 2 h, the fluorescence intensity of each system was determined using a Hitachi F-7000 fluorospectrometer (Tokyo, Japan) (excitation wavelengths: 488 nm and 633 nm).

Release kinetics assay: Eight parallel aliquots of the Anti-hTERT/GPX4-MnO_2_-NS probe solution (40 μg/mL) were prepared and incubated with GSH (10 mM) for varying durations (0, 0.5, 1.0, 1.5, 2, 2.5, 3, and 4 h), respectively. The fluorescence intensity of each reaction mixture was subsequently measured at its designated time point.

### 2.5. Cell Culture

Four cancer cell lines including human lung cancer cell line (A549), human cervical cancer cells (HeLa), human hepatocellular cancer cell line (HepG2), human colon cancer cell line (Caco-2), and one normal human hepatocyte cell line (HL-7702) were obtained from the Chinese Academy of Sciences Cell Bank (Shanghai, China). The cells were cultured in high-glucose Dulbecco’s Modified Eagle Medium (DMEM) or RPMI-1640 supplemented with 10% (*v*/*v*) fetal bovine serum (FBS; Gibco, Thermo Scientific, Waltham, MA, USA) and 1% antibiotics (Hyclone, Thermo Scientific, Waltham, MA, USA), at 37 °C in a humidified atmosphere of 5% CO_2_.

Cell washing was performed using Dulbecco’s Phosphate-Buffered Saline (DPBS) without calcium and magnesium (Ca^2+^/Mg^2+^-free; Invitrogen, Thermo Scientific, Waltham, MA, USA). Cell counting was performed using a cell counter.

### 2.6. In Situ Imaging of MnO_2_-NS Probes in A549, HeLa, HepG2, and Caco-2 Cells

Cancer cells (0.4 mL, 1 × 10^6^ cells/mL) were seeded into 20 mm confocal dishes and cultured for 24 h. An appropriate amount of the MnO_2_-NS probe stock solution was then added to each dish, resulting in a final MnO_2_-NS concentration of 30 μg/mL. After further incubation at 37 °C for 6 h, the culture medium was removed, and the cells were washed twice with PBS. Subsequently, an appropriate volume of PBS was added, and the cells were subjected to imaging using a confocal laser scanning microscope (Zeiss LSM880, Jena, Germany) with excitation wavelengths of 488 nm and 633 nm.

### 2.7. Cell Viability Assay

Four different cell lines (A549, HeLa, HepG2, and Caco-2) were seeded into 96-well plates (1 × 10^5^ cells per well) and cultured for 24 h. The cells were then treated with varying concentrations (0, 1, 5, 10, 20, 30, 40, and 50 μg/mL) of MnO_2_-NS probe or Control-MnO_2_-NS probe for 72 h. After treatment, the medium was removed, and MTT solution (0.5 mg/mL) was added to each well. Following incubation at 37 °C for 4 h, the medium was discarded, and 100 μL of DMSO was added to each well. The plate was then shaken at room temperature for 10 min to dissolve the formazan crystals formed by viable cells. The absorbance of each well was measured at 490 nm using a microplate reader (Biotek, Agilent, Santa Barbara, CA, USA). Relative cell viability was calculated as (*A*_Test_/*A*_Control_) × 100.

### 2.8. qRT-PCR Analysis of hTERT mRNA and GPX4 mRNA Expression

A549 and HeLa cells were seeded in 6-well plates and cultured for 24 h. Each cell line was divided into three experimental groups: a PBS group (Control), an MnO_2_-NS probe group, and a Control-MnO_2_-NS probe group. At specified time points, PBS or the corresponding probes (30 μg/mL) were added to the cell cultures. After the designated treatment periods (24 or 48 h), the culture medium was removed, and the cells were thoroughly washed with PBS. The cells were then trypsinized, counted, and subjected to RNA extraction using Trizol total RNA isolation reagent (TIANGEN, Beijing, China) according to the manufacturer’s instructions. cDNA synthesized using the QuantiNova Reverse Transcription Kit (Qiagen, Duesseldorf, Germany) was stored at −80 °C for subsequent qRT-PCR analysis. The mRNA expression levels of hTERT and GPX4 were determined by real-time PCR on a QuantStudio™ 5 System (Applied Biosystems, Foster, CA, USA) using SYBR Green Master Mix. The primers employed in this study are provided in Table 1. The relative expression level of hTERT or GPX4 mRNA was derived from the 2^−ΔΔCT^ method, normalized to GAPDH and to the level in untreated cells. All assays were carried out in triplicate. The thermal cycling conditions comprised an initial step at 95 °C for 30 s, then 35 cycles of 95 °C for 5 s, 58 °C for 30 s, and 72 °C for 20 s.

### 2.9. Quantification of Intracellular hTERT, GPX4, and Se-GPX Activity

A549 and HeLa cells were cultured and treated with the probes as described in Section 2.8. Following treatment, the cells were washed with PBS, detached with 0.02% EDTA, and subsequently lysed using a glass homogenizer. The concentration of hTERT in the cell lysates was quantified using a commercial hTERT ELISA kit. The GPX4 activity in the cell lysates was determined using a commercial GPX4 Fluorogenic Assay Kit (Amyjet Scientific, Wuhan, China). To determine Se-GPX activity, the total protein concentration in the lysates was first measured with a BCA protein assay kit. The enzymatic activity of Se-GPX was then evaluated using a glutathione peroxidase assay kit (NADPH method, Beyotime, Shanghai, China), following the manufacturer’s protocol.

### 2.10. Analysis of Intracellular GSH Levels

Following the protocol in Section 2.9, HeLa cells were cultured and exposed to the probes. At the indicated time points (24, 48, and 72 h), the treated cells were subjected to PBS washing and trypsin-mediated detachment. After collection by centrifugation, the cell pellets were incubated with 100 μL of RIPA-PMSF solution on ice for 30 min to achieve lysis. Quantification of intracellular GSH was performed using the DTNB method with a commercial GSH/GSSG assay kit (Beyotime, China).

### 2.11. Analysis of Intracellular ROS Levels

HeLa cells were seeded in 12-well plates at a density of 1 × 10^5^ cells per well and cultured for 24 h. The cells were then divided into three experimental groups: a PBS group (Control), an MnO_2_-NS probe group, and a Control-MnO_2_-NS probe group. At specified time points, PBS or the corresponding probes (30 μg/mL) were added to the cultures. After the designated treatment periods (24, 48, and 72 h), the culture medium was removed, and the cells were carefully washed with PBS. Subsequently, 2′,7′-dichlorofluorescein diacetate (DCF-DA) at a concentration of 10 μM was applied and incubated with the cells at 37 °C for 30 min. Following another PBS wash, the stained cells were collected, and intracellular ROS levels were analyzed using a flow cytometer (Guava easyCyte 6–2 L, Merck Millipore, Burlington, MA, USA).

### 2.12. Measurements of Intracellular LPO Levels

HeLa cells were cultured and treated with the probes as described in Section 2.9. After treatment (24, 48, and 72 h), the cells were washed with PBS and detached with trypsin. The harvested cells were then centrifuged, and the resulting pellets were lysed. Protein concentrations were measured using a BCA protein assay kit (Beyotime, China). Based on the protein quantification results, LPO levels—specifically malondialdehyde (MDA)—were assessed in each probe group and control group using a commercial lipid peroxidation MDA assay kit (Beyotime, China).

### 2.13. Statistical Analysis

All data were reported as the means ± standard deviation from three independent experiments. Statistical analyses were performed using the IBM SPSS Statistics 25 software package, and we assumed no significance at *p* > 0.05 (ns), low significance at *p* < 0.05 (*), medium significance at *p* < 0.01 (**) and high significance at *p* < 0.001 (***).

## 3. Results and Discussion

### 3.1. Preparation and Characterization of MnO_2_-NSs and Anti-hTERT/GPX4-DNA-MnO_2_-NS Probes

**Figure 2:**
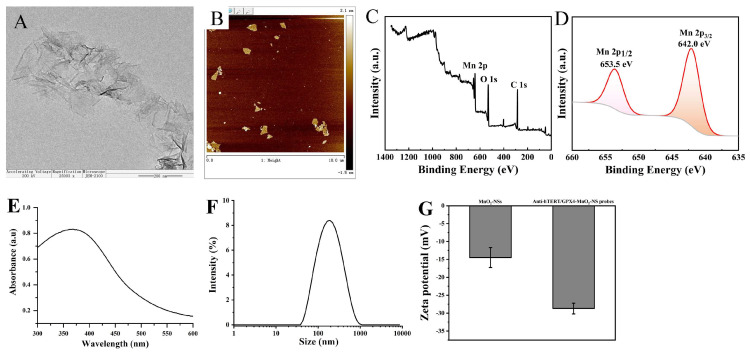
(**A**) TEM, (**B**) AFM images, (**C**,**D**) XPS characterization, (**E**) UV−vis absorption spectrum, and (**F**) size distribution of MnO_2_-NSs. (**G**) Zeta potential of MnO_2_-NSs and Anti-hTERT/GPX4-MnO_2_-NS probes.

The preparation of MnO_2_-NSs was carried out by oxidizing Mn^2+^ with H_2_O_2_ in the presence of TMA cations [16]. The morphological features, thickness, and hydrodynamic size distribution of the synthesized nanosheets were examined using transmission electron microscopy (TEM), atomic force microscopy (AFM), and dynamic light scattering (DLS), respectively. As revealed by TEM (Figure 2A) and AFM (Figure 2B), the MnO_2_-NSs displayed a typical two-dimensional nanosheet morphology. Based on the X-ray photoelectron spectroscopy (XPS) data, the Mn 2p orbital in MnO_2_-NSs exhibits two characteristic peaks: Mn 2p_1/2_ at 653.5 eV with an intensity of 0.80 a.u. and Mn 2p_3/2_ at 642.0 eV with an intensity of 0.95 a.u. (Figure 2C,D), where the binding energy separation of about 11.5 eV is typical of Mn^4+^ and indicates that manganese primarily exists in the +4 oxidation state, consistent with MnO_2_. This confirms that the material is MnO_2_-NSs with Mn in the +4 oxidation state. The UV–vis spectrum of the MnO_2_-NSs displayed a characteristic absorption maximum around 370 nm (Figure 2E), in agreement with previous literature [17], thus confirming the successful synthesis of the nanosheets. To enhance cellular uptake, the MnO_2_-NSs were ultrasonically fragmented into smaller nanoparticles (Figure 2F). The resulting fragments, with a hydrodynamic size ranging from 50 to 600 nm (mean diameter: 205 ± 10 nm), were then used as carriers for the Anti-hTERT-DNA/Anti-GPX4-DNA/MnO_2_-NS probes. Following the loading of antisense sequences, the zeta potential of MnO_2_-NSs decreased from −14.5 to −27.8 mV (Figure 2G), which confirmed the successful construction of the Anti-hTERT-DNA/Anti-GPX4-DNA/MnO_2_-NS probes.

### 3.2. Fluorescence Quenching and Recovery

It has been widely demonstrated that MnO_2_-NSs adsorb single-stranded DNA (ssDNA) and efficiently quench the fluorescence of DNA-conjugated labels (e.g., FAM, Cy3, Cy5) [15,17]. Since MnO_2_-NSs can be degraded by the elevated GSH in cancer cells into Mn^2+^, the ssDNA is released intracellularly, restoring the fluorescence signal. Based on this quenching-and-recovery mechanism, we confirmed the construction of the Anti-hTERT/GPX4-DNA-MnO_2_-NS probes by observing the initial fluorescence quenching upon probe assembly and the subsequent signal recovery upon GSH-triggered MnO_2_ dissolution.

**Figure 3:**
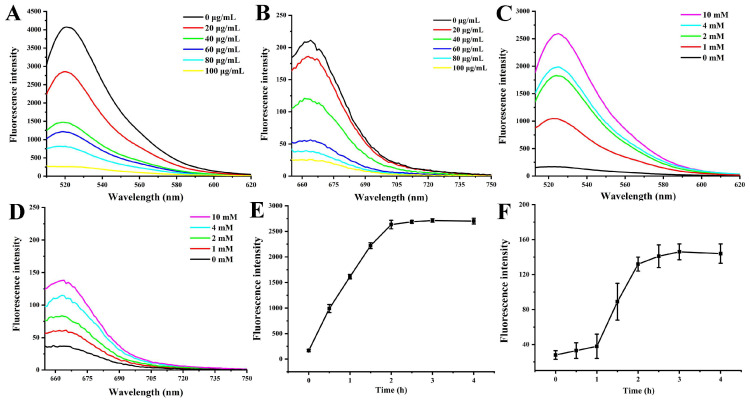
Fluorescence quenching of (**A**) Anti-hTERT-DNA (100 nM) and (**B**) Anti-GPX4-DNA (100 nM) strands was assessed upon their adsorption onto MnO_2_-NSs at increasing concentrations. (**C**,**D**) Fluorescence recovery of the Anti-hTERT/GPX4-MnO_2_-NS probes (40 μg/mL) was subsequently evaluated with the addition of increasing concentrations of GSH. (**E**,**F**) GSH-triggered release kinetics of the Anti-hTERT/GPX4-MnO_2_-NS probes (40 μg/mL) when mixed with GSH (10 mM). The values are expressed as the mean ± SE (*n* = 3).

Varying concentrations of MnO_2_-NSs were incrementally introduced into solutions containing the Anti-hTERT-DNA and Anti-GPX4-DNA strands. Following a 30 min incubation period, the extent of fluorescence quenching was measured to assess the adsorption efficiency of the MnO_2_-NSs towards the two anti-sense sequences. In the absence of MnO_2_-NSs, the FAM-labeled Anti-hTERT-DNA and Cy5-labeled Anti-GPX4-DNA strands, when excited at 488 nm and 633 nm, displayed characteristic fluorescence emission peaks at 525 nm and 663 nm, respectively. The results indicated a concentration-dependent decrease in fluorescence intensity with increasing amounts of MnO_2_-NSs. A complete quenching of both FAM and Cy5 fluorescence was achieved at an MnO_2_-NS concentration of 100 μg/mL (Figure 3A,B), which was therefore determined to be sufficient for effective probe adsorption. Consequently, to ensure optimal adsorption, the final MnO_2_-NS probe stock solution was prepared with an Anti-hTERT-DNA:Anti-GPX4-DNA:MnO_2_-NSs ratio of 100 nM:100 nM:100 μg/mL.

The intracellular concentration of GSH ranges from 2 to 10 mM, which is about 1000-fold higher than the extracellular level (2–10 μM) [18]. To validate that the Anti-hTERT/GPX4-MnO_2_-NS probes can effectively release their loaded antisense oligonucleotides upon encountering elevated GSH in cancer cells, we performed a fluorescence recovery assay by exposing the probes to 1–10 mM GSH. The results showed that treatment of the fully quenched MnO_2_-NS probes with GSH led to the concentration-dependent dismantling of the nanosheets. The degradation of the MnO_2_ carrier released the adsorbed DNA strands (Anti-hTERT-DNA and Anti-GPX4-DNA), as directly evidenced by the recovery of FAM and Cy5 fluorescence and the overall increase in signal intensity (Figure 3C,D). This fluorescence recovery confirms the effective, GSH-triggered degradation of the MnO_2_-NSs and the subsequent release of the therapeutic oligonucleotides. In addition, by monitoring the recovery of FAM and Cy5 fluorescence intensity, we analyzed the release kinetics of the antisense sequences from the probes in response to GSH. The results indicate that the Anti-hTERT/GPX4-MnO_2_-NS probes can effectively release the nucleic acid sequences within 2 h in a high-concentration GSH environment (10 mM) (Figure 3E,F).

### 3.3. In Situ Imaging of Cell Uptake of Anti-hTERT/GPX4-MnO_2_-NS Probes

**Figure 4:**
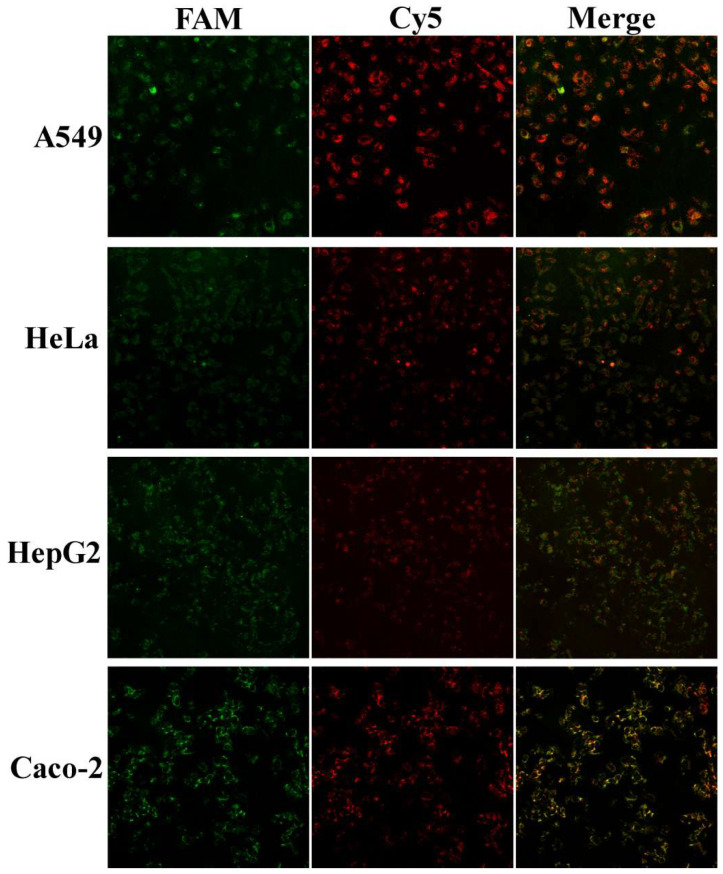
CLSM images of A549, HeLa, HepG2, and Caco-2 cells after treated with Anti-hTERT/GPX4-MnO_2_-NS probes (30 μg/mL) for 6 h.

We next evaluated the probe for in situ imaging in four cancer cell lines: A549 (lung), HeLa (cervical), HepG2 (liver), and Caco-2 (colon) by confocal laser scanning microscopy (CLSM). Following cellular uptake by endocytosis, the intracellular GSH microenvironment degrades the MnO_2_-NS carrier, releasing the FAM- and Cy5-labeled DNA strands and consequently recovering their fluorescence. Imaging after a 6 h incubation clearly revealed the characteristic dual-fluorescence signals within all tested cells (Figure 4), validating that intracellular GSH effectively degrades the probe and releases the functional nucleic acids into the cytoplasm. Although the fluorescence distribution pattern varied to some extent across cell types—most notably in HepG2 cells—the consistent presence of signal demonstrates successful cytoplasmic delivery of the anti-sense sequences.

### 3.4. In Vitro Anticancer Assay

Next, we employed the MTT assay to evaluate the inhibitory effect of MnO_2_-NS probes on cancer cell proliferation. As illustrated in Figure 4, when four types of cancer cells were treated with varying concentrations of Anti-hTERT/GPX4-MnO_2_-NS probes for 72 h, the viability of all cancer cell types declined progressively with increasing probe concentrations. Furthermore, at a probe concentration of 50 μg/mL, the survival rates of all four cancer cell lines dropped below 30%. These results demonstrate that the Anti-hTERT/GPX4-MnO_2_-NS probes possess significant anticancer activity in vitro. In contrast, under the same treatment conditions, Control-MnO_2_-NS probes exhibited only a minimal effect on cell survival. This confirms that the cell death-promoting effect of the Anti-hTERT/GPX4-MnO_2_-NS probes is attributable to the loaded Anti-hTERT and Anti-GPX4 DNA sequences.

**Figure 5:**
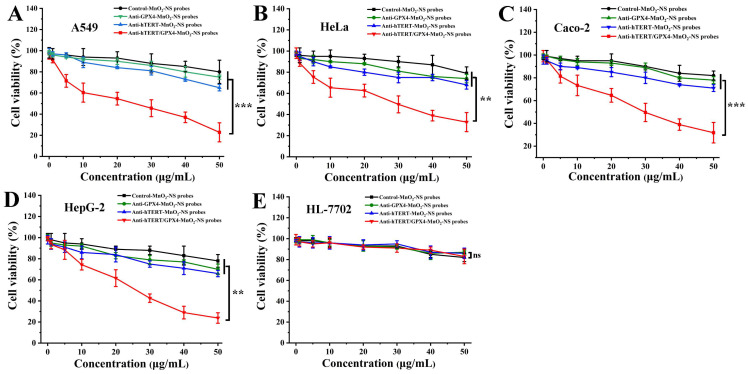
Effect of varying concentrations of different MnO_2_-NS probes on the survival rate of four cancer cell lines (**A**–**D**) and one normal cell line (**E**) after 72 h. Viability data are presented as the mean ± SE (*n* = 3); (**) *p* < 0.01 and (***) *p* < 0.001, significantly different from the control; (ns) *p* > 0.05, not significantly different from the control.

In previous studies, we investigated the induction of cancer cell death using gold nanoparticles as carriers, generating gold nanoprobes loaded solely with antisense oligonucleotides targeting hTERT mRNA [8]. The results indicated that after 72 h of treatment, these gold nanoprobes did affect the viability of HeLa and HepG2 cells, but the reduction in survival rate was less than 30%. Similarly, gold nanoprobes loaded with antisense oligonucleotides targeting GPX4 mRNA, prepared via the same method, showed almost no effect on the survival of A549, HeLa, and HepG2 cells [9]. These findings suggest that regulating either hTERT mRNA or GPX4 mRNA alone using antisense gene technology is insufficient to effectively induce cancer cell death. To further validate this, we also prepared Anti-hTERT-MnO_2_-NS and Anti-GPX4-MnO_2_-NS probes loaded exclusively with Anti-hTERT-DNA or Anti-GPX4-DNA, respectively. Under identical experimental conditions, the impact of these single-target MnO_2_-NS probes on cancer cell viability was consistent with previously reported data, showing only a modest reduction in survival rates (Figure 5) and further underscoring the limited efficacy of single-target inhibition. It should be noted that although the Anti-GPX4-MnO_2_-NS probes exhibited relatively poor efficiency in promoting cancer cell death, they still demonstrated certain anticancer activity in vitro compared to the previously reported gold nanoprobes targeting GPX4 mRNA [9]. This observed activity may be attributed to the presence of the MnO_2_-NS carrier itself. We propose that a synergistic effect is likely at play, where the down-regulation of GPX4 by Anti-GPX4-DNA works in concert with the depletion of intracellular GSH by the MnO_2_-NSs, collectively contributing to the observed pro-death effect. In contrast to their effects on cancer cells, all MnO_2_-NS probes exhibited substantially lower cytotoxicity against normal hepatocytes (HL-7702) (Figure 5E). The three antisense-loaded MnO_2_-NS probes showed comparable cell-killing effects to the control probes in normal cells. Given the low basal expression of hTERT and GPX4, together with the relatively lower GSH content in HL-7702 cells, these results suggest that the antisense-loaded MnO_2_-NS probes exerts selective cytotoxicity toward cancer cells.

These findings suggest that the Anti-hTERT/GPX4-MnO_2_-NS probes hold promising potential as a high-efficacy, low-toxicity anticancer agent. Their straightforward preparation further underscores a strong prospect for clinical translation. This advantage becomes particularly evident when compared to many existing strategies. For instance, achieving comparable levels of induced cell death with previously reported hTERT/GPX4-targeted therapies [19,20] or MnO_2_-based nanomedicines [10] often necessitates more complex synthetic procedures or highly intricate functional components. Such complexity typically results in lower batch-to-batch reproducibility and higher manufacturing costs. Conversely, simpler systems reported elsewhere [8,9] tend to demonstrate lower efficacy in promoting cell death, failing to achieve a robust anticancer outcome.

### 3.5. Analysis of Relative mRNA Levels and Protein Expression of hTERT and GPX4 in Cells

**Figure 6:**
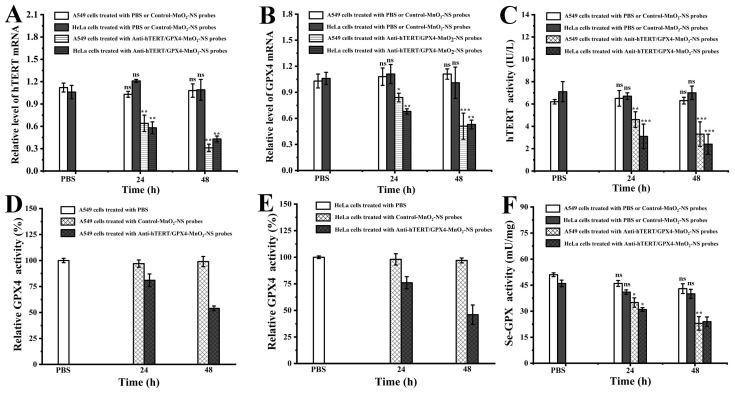
Determination of (**A**) hTERT and (**B**) GPX4 mRNA levels by RT-PCR in A549 and HeLa cells following treatment with PBS, Control-MnO_2_-NS probes (30 μg/mL), or Anti-hTERT/GPX4-MnO_2_-NS probes (30 μg/mL) for 24 or 48 h. Analysis of (**C**) hTERT activity, (**D**,**E**) relative GPX4 activity, and (**F**) Se-GPX activity in A549 and HeLa cells following treatment with PBS, Control-MnO_2_-NS probes (30 μg/mL), or Anti-hTERT/GPX4-MnO_2_-NS probes (30 μg/mL) for 24 or 48 h. The values are expressed as the mean ± SE (*n* = 3); (*) *p* < 0.05, (**) *p* < 0.01, and (***) *p* < 0.001, significantly different from the control (PBS); (ns) *p* > 0.05, not significantly different from the control (PBS).

The intracellular relative mRNA levels of hTERT and GPX4 were quantified using RT-PCR. A549 and HeLa cells were selected as experimental models. As expected, the results indicated that treatment with Anti-hTERT/GPX4-MnO_2_-NS probes led to a significant down-regulation of hTERT and GPX4 mRNA. Moreover, the down-regulatory effect became more pronounced with prolonged exposure time. In contrast, no decrease in hTERT or GPX4 mRNA levels was observed in cells treated with Control-MnO_2_-NS probes (Figure 6A,B). These findings demonstrate that MnO_2_-NS can effectively serve as a carrier for delivering antisense oligonucleotides targeting hTERT and GPX4 mRNA, thereby reducing their intracellular expression. Subsequently, we assessed the protein expression levels of hTERT and GPX4 using specific activity assay kits. Due to the activity of total GPX containing selenium (Se-GPX) is consistent with the expression of GPX4, here the activity of total GPX containing selenium (Se-GPX) were also determined using a glutathione peroxidase assay kit with the NADPH method. The studies revealed that hTERT, GPX4, and Se-GPX activities were significantly down-regulated following treatment with Anti-hTERT/GPX4-MnO_2_-NS probes (Figure 6C–F).

### 3.6. Analysis of Intracellular GSH Level, ROS Level, and MDA Content

**Figure 7:**
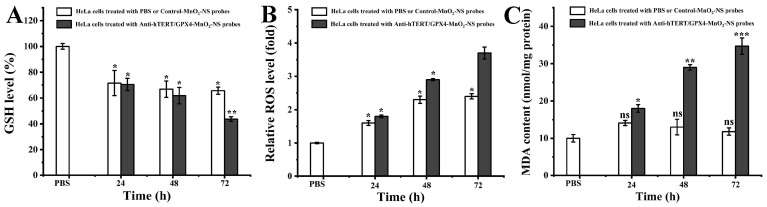
Analysis of intracellular (**A**) GSH levels, (**B**) relative ROS levels, and (**C**) MDA contents of HeLa cells following treatment with PBS, Control-MnO_2_-NS probes (30 μg/mL), or Anti-hTERT/GPX4-MnO_2_-NS probes (30 μg/mL) for 24, 48, or 72 h. The values are expressed as the mean ± SE (*n* = 3); (*) *p* < 0.05, (**) *p* < 0.01, and (***) *p* < 0.001, significantly different from the control; (ns) *p* > 0.05, not significantly different from the control (PBS).

MnO_2_ nanomaterials have been demonstrated to be reduced to Mn^2+^ by the glutathione reductase cycle within cancer cells. This process depletes GSH and, by consuming NADPH, imposes substantial stress on the cellular antioxidant system, ultimately leading to a decrease in overall intracellular GSH levels [10]. This mechanism is considered a primary factor underlying the cytotoxicity of MnO_2_ nanomaterials. After confirming the cytotoxic effects of MnO_2_-NSs on four cancer cell lines via the MTT assay, we subsequently measured changes in intracellular GSH levels following treatment with two types of MnO_2_-NS probes using the DTNB method. The results indicated that both Control-MnO_2_-NS probes and Anti-hTERT/GPX4-MnO_2_-NS probes induced a similar degree of GSH depletion after 48 h of incubation (Figure 7A). However, after 72 h, the Anti-hTERT/GPX4-MnO_2_-NS probes provoked a more pronounced decline in GSH content. We hypothesize that this enhanced depletion is likely a consequence of simultaneously consuming the GSH reserve and disrupting the cellular GSH biosynthesis system, which is caused by probe-induced cell death [21,22].

Tumor cells exhibit elevated levels of ROS compared to normal cells [23,24]. To maintain redox homeostasis, these cells often up-regulate several antioxidants—such as GSH, glutathione peroxidase (GPX), and ascorbic acid—to scavenge intracellular ROS [10]. Therefore, when the levels of GSH and GPX are down-regulated, a consequent increase in ROS levels would be expected. To test this, we measured intracellular ROS levels in HeLa cells treated with Control-MnO_2_-NS probes or Anti-hTERT/GPX4-MnO_2_-NS probes for different durations, using the fluorescent probe DCF-DA. As shown in Figure 7B, Control-MnO_2_-NS probes indeed induced a certain increase in intracellular ROS. In contrast, the Anti-hTERT/GPX4-MnO_2_-NS probes led to a more pronounced rise in ROS, which further intensified with prolonged treatment time. This enhanced effect is likely attributable to the combined action of MnO_2_-NS–mediated GSH depletion and GPX4 down-regulation induced by the anti-sense oligonucleotide.

Numerous studies have demonstrated that down-regulation of hTERT alone can lead to telomere shortening and activation of the DNA damage response, ultimately triggering the intrinsic apoptotic pathway [25,26]. Additionally, some small-molecule hTERT inhibitors have been reported to induce not only apoptosis but also ferroptosis in cancer cells [2]. In contrast, inhibiting GPX4 or depleting GSH alone may not reliably induce ferroptosis in cancer cells [9]. Given that ferroptosis is characterized by elevated cellular ROS and LPO—and since intracellular ROS levels had already been measured—we next assessed LPO by measuring MDA content. This allowed us to evaluate the likelihood of ferroptosis in cancer cells treated with Anti-hTERT/GPX4-MnO_2_-NS probes. The results revealed that Control-MnO_2_-NS probes had a minimal effect on MDA levels in HeLa cells (Figure 7C). In contrast, treatment with Anti-hTERT/GPX4-MnO_2_-NS probes significantly increased MDA content in a time-dependent manner. Therefore, combined with the MTT assay results (Figure 5), these findings suggest that simultaneous down-regulation of hTERT and GPX4 mRNA along with GSH depletion—leading to reduced activities of hTERT and GPX4, ROS accumulation, and elevated lipid peroxidation—likely promotes enhanced antitumor efficacy.

## 4. Conclusions

We constructed a MnO_2_-NS probe for the co-delivery of antisense oligonucleotides against hTERT and GPX4 mRNA. The probe’s assembly and cellular internalization were verified via fluorescence quenching/recovery and confocal imaging. Functionally, the probe down-regulated target mRNA and protein activity. Cancer cell death was enhanced through a synergistic effect: the gene silencing mechanism, combined with MnO_2_-NS-mediated GSH depletion, elevated ROS and LPO. This work establishes the MnO_2_-NS-based co-delivery system as a potent and promising anticancer strategy. However, numerous challenges remain when applying this system for in vivo anticancer therapy, such as tumor targeting and the leakage of the drug during systemic circulation. Therefore, there is considerable room for optimization of the system. For instance, modifying the surface of MnO_2_-NSs with hyaluronic acid, a tumor-targeting component, could enhance tumor-specific targeting and reduce the leakage of bare MnO_2_-NSs during circulation. The specific effects of such modifications will be further investigated in subsequent studies.

## Figures and Tables

**Table 1:** DNA sequence list.

Names	Sequences
Anti-hTERT-DNA	5′-TCC ATG TTC ACA ATC GGC C-FAM-3′
Anti-GPX4-DNA	5′-GAT ACG CTG AGT GTG GTT T-Cy5-3′
Control-DNA	5′-CCA GAT CAA GAT CCA TTG A-3′
hTERT-F-primer	5′-CGG AAG AGT GTC TGG AGC AA-3′
hTERT-R-primer	5′-CAC GAC GTA GTC CAT GTT CA-3′
GPX4-F-primer	5′-AGA GAT CAA AGA GTT CGC CGC-3′
GPX4-R-primer	5′-TCT TCA TCC ACT TCC ACA GCG-3′
GAPDH-F-primer	5′-CTC AGA CAC CAT GGG GAA GGT GA-3′
GAPDH-R-primer	5′-ATG ATC TTG AGG CTG TTG TCA TA-3′

## Data Availability

The original contributions presented in this study are included in the article. Further inquiries can be directed to the corresponding author.
